# Esketamine in a Patient With Major Depressive Disorder and Polysubstance Use: A Case Report

**DOI:** 10.7759/cureus.93876

**Published:** 2025-10-05

**Authors:** Abdelazim Ali, Nahida Ahmed, Sona Varghese

**Affiliations:** 1 Psychiatry, Maudsley Health Services, Dubai, ARE; 2 Psychiatry, Sakina, Abu Dhabi, ARE

**Keywords:** clinical case report, craving reduction, esketamine, esketamine intranasal, glutamatergic modulation, major depressive disorder (mdd), methamphetamine use disorder, opioid use disorder (oud), polysubstance use disorder, treatment-resistant depression

## Abstract

Major depressive disorder (MDD) frequently co-occurs with substance use disorders (SUDs), leading to poorer outcomes and limited options. Esketamine is approved for treatment-resistant depression (TRD); its effects on craving and abstinence in SUD are less explored. A 41-year-old man with recurrent MDD (suicidal ideation) and polysubstance use (daily morphine/prescription opioids; intermittent methamphetamine) failed multiple antidepressants and engaged in opioid-seeking behavior. Intranasal esketamine was initiated adjunctive to the ongoing oral antidepressant. At one month, depressive symptoms and craving markedly improved. At two months, he achieved abstinence from opioids and methamphetamine. At four months, he returned to work and started a business. At six months, he remained euthymic and abstinent. The Patient Health Questionnaire-9 (PHQ-9) scores dropped progressively from 24 at baseline to 4 at six months. Tolerability was good (transient dizziness, mild dissociation). Beyond rapid antidepressant effects, esketamine may reduce craving via glutamatergic modulation and synaptic plasticity. In conclusion, esketamine may offer dual benefits in TRD with comorbid SUD; however, controlled studies are needed.

## Introduction

Major depressive disorder (MDD) and substance use disorder (SUD) commonly co-occur, with estimates suggesting that up to 50% of individuals with SUD meet the criteria for MDD. This comorbidity is associated with higher relapse rates, suicide risk, and functional impairment. Conventional antidepressants often fail to achieve remission in this population, partly due to neurobiological overlap between mood regulation and reward pathways. A meta-analysis published in 2020 revealed that SUDs in MDD are highly prevalent and rates have not changed over time [1]. This high prevalence suggests the need to develop better treatment and prevention options.

Esketamine, the S-enantiomer of ketamine, is an N-methyl-D-aspartate (NMDA) receptor antagonist approved for treatment-resistant depression (TRD). Its rapid antidepressant effects are mediated by increased α-amino-3-hydroxy-5-methyl-4-isoxazolepropionic acid (AMPA) throughput, brain-derived neurotrophic factor (BDNF) release, and synaptogenesis in mood-regulating circuits. Early studies also suggest potential benefits in reducing craving and promoting abstinence in alcohol, cocaine, and opioid use disorders. Given that depressive symptoms are highly prevalent among individuals with alcohol disorders and SUDs and that esketamine has been shown to produce sustained improvements in depressive symptoms and overall functioning [2], it is plausible that esketamine could serve as a promising therapeutic option in the management of SUDs, particularly in cases where comorbid depression is present.

The primary focus of this report is on the management approach and therapeutic efficacy of esketamine in the setting of comorbid polysubstance use. Our findings suggest that esketamine may be particularly beneficial for patients with polysubstance use disorders when MDD is the principal associated diagnosis. We suggest that the use of esketamine may be particularly effective in patients with polysubstance use when depression is the primary associated diagnosis. 

## Case presentation

Patient history

The patient is a 41-year-old married man with a 15-year history of MDD and chronic polysubstance use. He initially began opioids after a musculoskeletal injury and progressed to morphine and codeine-containing analgesics, with intermittent methamphetamine use (approximately weekly binges escalating to several times per week prior to treatment). He had multiple prior psychiatric admissions for severe depression with suicidal ideation.

He had been treated with multiple antidepressants, including selective serotonin reuptake inhibitors (SSRIs) (sertraline, paroxetine), serotonin-norepinephrine reuptake inhibitors (SNRIs) (venlafaxine, duloxetine), bupropion, mirtazapine, and vortioxetine, as well as antipsychotic augmentation (aripiprazole, quetiapine) with limited benefit. He engaged in "doctor shopping" for painkillers and had a strained marital relationship. No psychosis or significant medical comorbidity was noted. Family support was present but strained by his substance use.

Mental state examination (baseline)

On examination, the patient appeared disheveled and tired. His speech was slow and of low volume. He described his mood as "hopeless," with a restricted affect. Suicidal ideation was present, though no delusional content was elicited. Cognitive functions were intact, and insight was assessed as partial.

Intervention

Given the coexistence of severe depression and opioid dependence, intranasal esketamine was initiated at 56 mg in the first dose. After two days, 84 mg was given, and then after that, the same dose of 84 mg was given twice weekly for four weeks and then 84 mg weekly, under supervision, while maintaining his baseline antidepressant. Each session was monitored for vital signs, dissociation, and adverse effects. After one month, depressive symptoms and craving markedly improved. At two months, he achieved abstinence from opioids and methamphetamine, confirmed by urine drug screens (UDS). At four months, he returned to work and started a business. At six months, he remained euthymic, abstinent, and fully functional. The Patient Health Questionnaire-9 (PHQ-9) scores dropped from 24 at baseline to 4 at six months. Tolerability was good (transient dizziness, mild dissociation) (Table 1).

**Table 1:** Clinical course and results PHQ-9: Patient Health Questionnaire-9

Time point	PHQ-9	Craving (0-10)	Substance use	Functional status
Baseline	24 (severe)	9/10	Daily opioids, intermittent meth	Unemployed
Month 1	17 (moderate)	5/10	Significant reduction	Improved energy, sleep
Month 2	6 (mild)	1/10	Abstinent	Initiated social activity
Month 4	5 (mild)	0/10	Abstinent	Started own business
Month 6	4 (minimal)	0/10	Sustained abstinence	Fully functional

Treatment was well tolerated, with transient dizziness and mild dissociation resolving within one hour. No cardiovascular instability or psychotic symptoms were observed.

## Discussion

This case illustrates esketamine's potential to simultaneously treat TRD and reduce substance craving, leading to sustained abstinence and functional recovery. 

Antidepressant effect

The effect of esketamine in improving depressive symptoms has been demonstrated in multiple studies. In a phase 3, open-label, single-arm long-term extension study (SUSTAIN-3) conducted from June 2016 to December 2022, depressive symptoms and functioning improved during the first four weeks of treatment and were sustained for up to 6.5 years with intermittently dosed esketamine plus daily antidepressant [3]. Our patient's PHQ-9 dropped from 24 to 6 within two months, consistent with rapid-onset effects reported in the literature.

Effect on craving and abstinence

Ketamine has shown promise in reducing relapse in alcohol use disorder and facilitating abstinence. A double-blind placebo-controlled trial showed that ketamine increased the number of days of abstinence from alcohol at three and six months compared with placebo [4]. Ketamine may also support abstinence in alcohol and other substances by alleviating depressive symptoms, especially during the high-risk relapse period after detoxification [5].

Mechanistic considerations

Esketamine may restore prefrontal control over limbic reward circuits, normalize dopaminergic signaling, and enhance neuroplasticity, allowing patients to re-engage in healthier behaviors. Improvement in mood likely synergized with craving reduction, breaking the cycle of self-medication [5].

Clinical implications

Patients with TRD and SUD represent a particularly high-risk group and need comprehensive interventions beyond pharmacology, such as psychotherapy and relapse prevention strategies, with close monitoring to minimize misuse risk. 

To our knowledge, this is the first reported case of esketamine in TRD with polysubstance use from the Middle East. 

## Conclusions

Esketamine produced rapid and sustained remission of both depression and polysubstance use in this patient, allowing complete functional recovery. This case supports the need for prospective and controlled trials investigating esketamine's dual role in mood and addiction treatment with integrated psychosocial interventions.
